# Optimizing Opioid Use in Pain Management: A Comprehensive Review of Clinical Benefits, Risks, and Dependence

**DOI:** 10.3390/healthcare14040457

**Published:** 2026-02-11

**Authors:** Francisco Josué Cordero-Pérez, Manuel Jesús Pérez-Baena, Nuria Pina Rubiralta, Anselma Fernández-Testa, Marina Holgado-Madruga

**Affiliations:** 1 Department of Internal Medicine, Complejo Asistencial de Zamora, 49022 Zamora, Spain; 2 Institute for Biomedical Research of Salamanca (IBSAL), P.º de San Vicente, 58, 182, 37007 Salamanca, Spain; mjperezbaena@usal.es (M.J.P.-B.); 3 Institute of Molecular and Cellular Biology of Cancer (IBMCC-CIC), University of Salamanca/Spanish National Research Council (CSIC), Campus Miguel de Unamuno, 37007 Salamanca, Spain; 4 Palliative Care Unit, Hospital Los Montalvos, Complejo Asistencial Universitario de Salamanca (CAUSA), 37115 Salamanca, Spain; 5 Emergency Department, Complejo Asistencial de Zamora, 49022 Zamora, Spain; 6 Department of Physiology and Pharmacology, University of Salamanca, 37007 Salamanca, Spain; 7 Institute of Neurosciences of Castilla y León (INCyL), University of Salamanca, C/Pintor Fernando Gallego 1, 37007 Salamanca, Spain; 8 Virtual Institute for Good Health and Well-Being (GLADE), European Campus of City Universities (EC2U), 86073 Poitiers, France

**Keywords:** opioids, pain management, chronic pain, multimodal therapy, dependence

## Abstract

**Highlights:**

**What are the main findings?**

- Opioids are essential for managing severe acute and cancer-related pain; however, their role in chronic non-cancer pain remains controversial. Concerns regarding dependence, tolerance, and misuse have intensified during the opioid crisis.
- This narrative review synthesizes recent evidence on opioid pharmacology, clinical applications, dependence risk factors, and the influence of genetic, psychological, and social determinants of opioid use. It highlights emerging safer therapies, opioid rotation, and multimodal strategies that combine pharmacological and nonpharmacological approaches.
- Health policies should promote equitable access to essential opioids while minimizing their misuse through individualized prescribing, regular patient monitoring, and the integration of non-pharmacological interventions. Reducing prescription bias and addressing disparities are critical for improving global pain care.

**What are the implications of the main findings?**

- Clinicians should prioritize a patient-centered, multimodal approach to pain management, reserving opioids for clearly indicated situations and combining them with non-pharmacological therapies to reduce long-term risks and improve functional outcomes.
- Health systems and policymakers should implement prescribing strategies that balance access and safety, including risk stratification, regular reassessment, and efforts to reduce social and racial disparities in opioid availability and pain treatment efficacy.

**Abstract:**

Effective pain management is central to anesthesia, critical care, and perioperative medicine, and opioids remain essential agents for moderate-to-severe pain despite ongoing concerns regarding their safety and misuse. This narrative review synthesizes the current knowledge on opioid mechanisms, clinical indications, safety considerations, and evolving strategies aimed at optimizing their use. Opioids exert their analgesic effects primarily through μ-, δ-, and κ-opioid receptors, which modulate central and peripheral nociceptive pathways. They maintain a well-established role in acute postoperative and cancer-related pain, whereas their use in chronic non-cancer pain remains controversial. Contemporary evidence suggests that physiological dependence and addiction are less frequent in appropriately selected and monitored patients, although the risk increases in the presence of psychological comorbidity, prior substance use, or adverse social determinants of health. Unequal access, prescribing variability, and persistent disparities further complicate global opioid management strategies. Recent advances, including partial agonists such as buprenorphine, dual-mechanism agents such as tapentadol, individualized titration, opioid rotation, and the integration of multimodal analgesia, support safer and more tailored prescribing. Non-pharmacological interventions, including behavioral and physical therapies, increasingly complement pharmacological strategies to minimize opioid exposure and improve functional outcomes. Clinicians must balance analgesic efficacy with adverse effects, such as tolerance, opioid-induced hyperalgesia, sedation, and respiratory depression, particularly in perioperative and critically ill populations. Opioids remain indispensable for selected indications but should be incorporated into a comprehensive, patient-centered, multimodal analgesic approach that prioritizes safety, ongoing reassessment, and individualized risk mitigation.

## 1. Introduction

Pain management is a fundamental pillar of contemporary medical care that addresses a universal and deeply personal human experience. The International Association for the Study of Pain (IASP) defines pain as “an unpleasant sensory and emotional experience associated with, or resembling that associated with, actual or potential tissue damage,” reflecting its complexity and subjectivity [1]. Pain is not merely a physiological response to injury; it also encompasses emotional distress, cognitive appraisal, and psychological suffering [2,3]. Its complexity is reflected in models such as Melzack and Casey’s, which involve sensory, affective, and cognitive dimensions [4], and the notion of “total pain,” which encompasses physiological, affective, sociocultural, behavioral, cognitive, and sensory components [5].

Pain affects more than one-third of the global population, with chronic pain affecting approximately 10% of the population each year. In Europe, nearly 20% of adults live with chronic pain, which imposes a heavy burden on health systems and economies [6,7,8,9,10].

Despite their long-standing therapeutic role, from early opium use to modern synthetic and semi-synthetic agonists, opioids remain essential for managing moderate-to-severe pain in anesthesiology, perioperative care, critical care, and palliative medicine. Their introduction has transformed the treatment of acute surgical pain, trauma, and cancer-related pain; however, their benefits are accompanied by substantial safety concerns. The U.S. opioid crisis underscores the consequences of excessive prescribing and inadequate monitoring. In sharp contrast, many low- and middle-income countries still lack even minimal access to opioid analgesia, leaving millions of patients with untreated pain owing to regulatory barriers, misinformation, and systemic inequities [11]. These opposing realities highlight the ongoing challenge of ensuring appropriate access while mitigating the risks of misuse, dependence, and overdose.

Accordingly, the objective of this narrative review is to provide a comprehensive and integrated examination of opioid use, addressing both their indispensable role in effective pain management and the challenges associated with their inappropriate use and potential for dependence. By synthesizing the current evidence on pharmacology, clinical indications, safety considerations, and evolving strategies, this review aims to support safer, rational, and context-sensitive opioid prescribing in contemporary clinical practice.

## 2. Methods

We conducted a narrative review to synthesize contemporary evidence on the mechanisms, clinical indications, risks, and evolving strategies for safer and individualized opioid use in the management of acute, chronic non-cancer, and cancer pain. A prespecified protocol outlining the objectives, sources, eligibility criteria, and synthesis plan guided its conduct, and reporting followed the best practice recommendations for narrative reviews. Literature searches were performed in MEDLINE (via PubMed), Embase, the Cochrane Library, Web of Science Core Collection, and Scopus, covering the period from 1 January 1980, to 30 June 2025.

To ensure comprehensive coverage, we also examined clinical guidelines and policy documents from authoritative bodies, including the World Health Organization (WHO), the Centers for Disease Control and Prevention (CDC), the National Institute for Health and Care Excellence (NICE), the European Society for Medical Oncology (ESMO), and the American Society of Clinical Oncology (ASCO), as well as grey literature from reputable reports and commission statements. ClinicalTrials.gov was screened for recently completed trials relevant to tapentadol and buprenorphine for pain management.

Search details and eligibility criteria are reported to improve transparency and reproducibility. The search combined MeSH terms (where available) and free-text term related to pain, opioid therapy (including specific drugs), effectiveness, adverse events, misuse/dependence, opioid-induced hyperalgesia, overdose, and tapering/discontinuation. Reference lists of key reviews and guidelines were also hand-searched. Included were human studies and evidence syntheses on opioid use for pain (acute, chronic non-cancer, or cancer), including trials, observational studies, systematic reviews/meta-analyses, guidelines, and policy documents reporting clinically relevant outcomes. Excluded were small case reports/series, editorials or opinion pieces without data, non-peer-reviewed sources, and studies focused only on perioperative anesthesia without pain outcomes. Full electronic search strings for each database are reported in Supplementary Table S1, and a brief study-selection flow diagram is provided in Supplementary Figure S1.

Quality appraisal was performed for all included sources: systematic reviews were evaluated using AMSTAR-2, randomized controlled trials using the Cochrane Risk of Bias 2 tool, observational studies using ROBINS-I or CASP checklists, and clinical guidelines using AGREE II. Evidence was synthesized thematically across five predefined domains: (1) mechanisms and pharmacology; (2) clinical indications and effectiveness in acute, chronic non-cancer, and cancer pain; (3) risks, including dependence, misuse, overdose, and opioid-induced hyperalgesia, and discontinuation; (4) optimization strategies, such as dose titration, opioid rotation, and the use of tapentadol or buprenorphine; and (5) policy and equity considerations. Quantitative estimates (e.g., prevalence ranges, number needed to treat, odds ratios) were reported descriptively when clinically relevant.

To aid in clinical interpretation, the evidence and recommendations were pragmatically graded using the GRADE framework. Certainty was rated as high, moderate, low, or very low, and the strength of recommendation was rated as strong, conditional/weak, or against. Grading was applied to clinical questions rather than individual studies, consistent with the narrative review methodology. Table 1 summarizes the GRADE classification of evidence certainty and recommendation strength.

## 3. Neurobiology and Physiology of Pain Relevant to Clinical Practice

**Figure 1:**
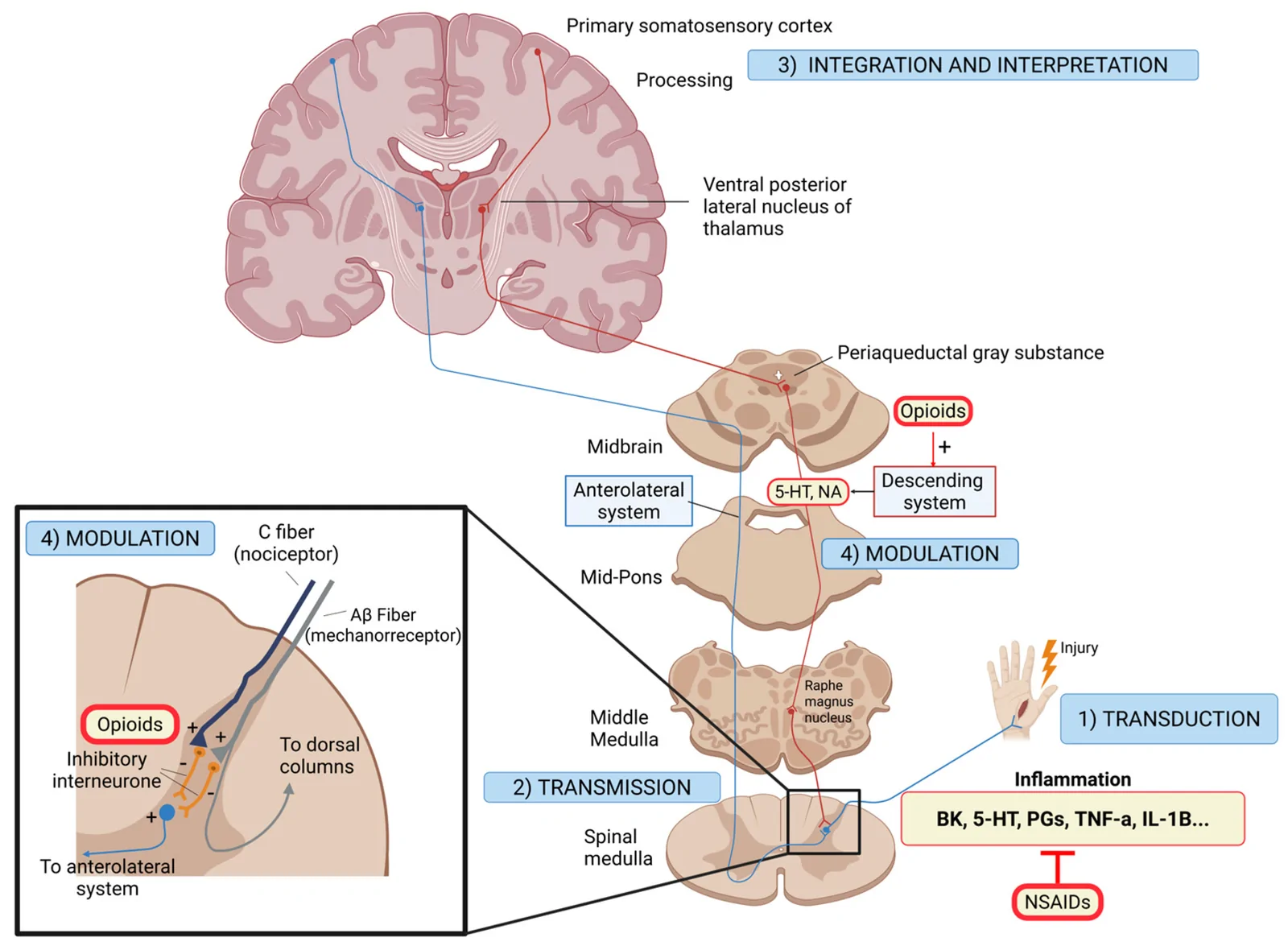
Pain Processing and Descending Inhibitory Control Mechanisms. Pain perception involves transduction, transmission, modulation, and perception, with endogenous opioids (enkephalins, endorphins, and dynorphins) inhibiting nociceptive activity and reducing pain signals at the spinal cord and brain levels. It is processed through ascending pathways (spinothalamic and spinoreticular tracts) and descending modulation via the PAG, RVM, and locus coeruleus. Created in BioRender. Cordero, J. (2026) https://BioRender.com/m41i501, accessed on 5 February 2026.

Pain can be categorized into nociceptive, neuropathic, inflammatory, and functional types, each with distinct underlying mechanisms. Contemporary pain classifications define nociplastic pain as a distinct pain mechanism characterized by altered central pain processing in the absence of clear tissue or nerve damage and is often poorly responsive to opioids [12] (Supplementary Table S2). The pain experience typically involves four key processes: transduction, transmission, modulation, and pain perception. Nociceptors detect harmful stimuli and send signals through the spinal cord to the brain (Figure 1) [13]. Peripheral and central sensitization can enhance pain, contributing to chronic conditions such as hyperalgesia and allodynia [14]. These processes involve inflammatory mediators, ion channel changes, NMDA receptor activity, and glial cell responses [15,16].

**Figure 2:**
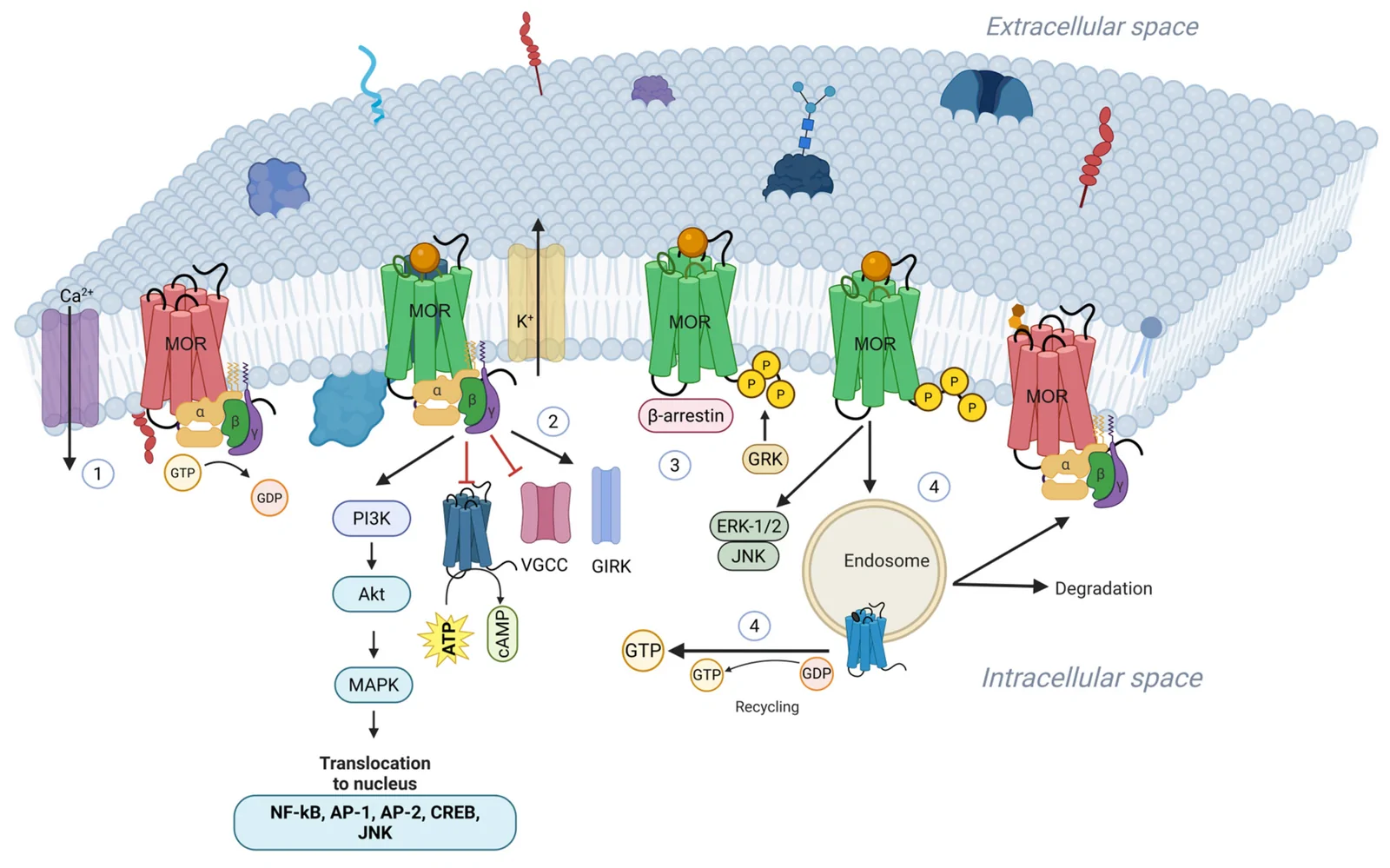
Opioid mechanism of action. The endogenous opioid system consists of four G protein-coupled receptors (GPCRs): mu (MOPR), delta (DOPR), kappa (KOPR), and nociceptin (NOPR) along with four primary families of endogenous opioid ligands: β-endorphins, enkephalins, dynorphins, and nociceptin/orphanin FQ. These receptors and peptides are widely distributed across the nervous system, particularly in the pain pathways. Upon activation by endogenous or exogenous agonists, opioid receptors couple to inhibitory G proteins (Gαi and Gαo), leading to the dissociation of G protein subunits (Gα and Gβγ), which modulate intracellular signaling cascades to suppress the neural activity. This inhibition occurs through the suppression of adenylate cyclase (AC), resulting in reduced cyclic AMP (cAMP) levels and blockade of voltage-gated calcium channels (VGCCs), which ultimately reduces neurotransmitter release. Consequently, synaptic vesicle fusion in presynaptic neurons is diminished, thereby dampening pain signal transmission. Furthermore, in dorsal root ganglion (DRG) neurons, prolonged agonist exposure can cause co-internalization of opioid receptors and N-type calcium channels, further attenuating nociceptive signaling in the central nervous system. Created in BioRender. Cordero, J. (2026) https://BioRender.com/t1w5clp, accessed on 5 February 2026.

Modulation occurs through descending inhibitory systems from brain regions such as the periaqueductal gray (PAG), rostroventral medulla (RVM), and locus coeruleus [17,18,19]. These systems rely on endogenous opioids—β-endorphins, enkephalins, dynorphins, and nociceptin/orphanin FQ—that act on specific receptors (MOPR, DOPR, KOPR, NOPR) [20,21]. Activation of these G protein-coupled receptors reduces neuronal excitability, inhibits neurotransmitter release, and promotes analgesia [19,22] (Figure 2 and Supplementary Table S3).

Exogenous opioids mimic these endogenous ligands and are powerful tools for managing moderate-to-severe pain [23]. Their effectiveness depends on the receptor affinity, metabolic pathways, and genetic variations. Factors such as liver metabolism, genetic polymorphisms (e.g., CYP2D6 and CYP3A4) [24,25], and drug transporters (e.g., P-glycoprotein/MDR1) can also play a role. These variables contribute to differences in efficacy, side effects [26,27], and risk of toxicity [25].

Despite their utility, opioids pose risks, such as tolerance, dependence, and addiction [21]. According to national NSDUH/SAMHSA surveys, the opioid crisis in the U.S. has revealed the dangers of overprescription: in 2017, over 11 million Americans misused opioids, with two million developing addiction [28]. Meanwhile, global access remains inequitable, with low- and middle-income countries—where severe pain is widespread—receiving less than 1% of the estimated need [11].

### Revisiting the WHO Analgesic Ladder: Historical Perspective and Limitations

The 1986 WHO cancer pain relief guidelines addressed pain management in patients with cancer through a stepwise approach emphasizing analgesics, particularly opioids. These guidelines have enhanced the global understanding of cancer pain management, especially in areas with restricted access to strong opioids. However, limitations include inconsistent implementation and the need for updates to incorporate newer pharmacological and interventional options [29,30].

**Figure 3:**
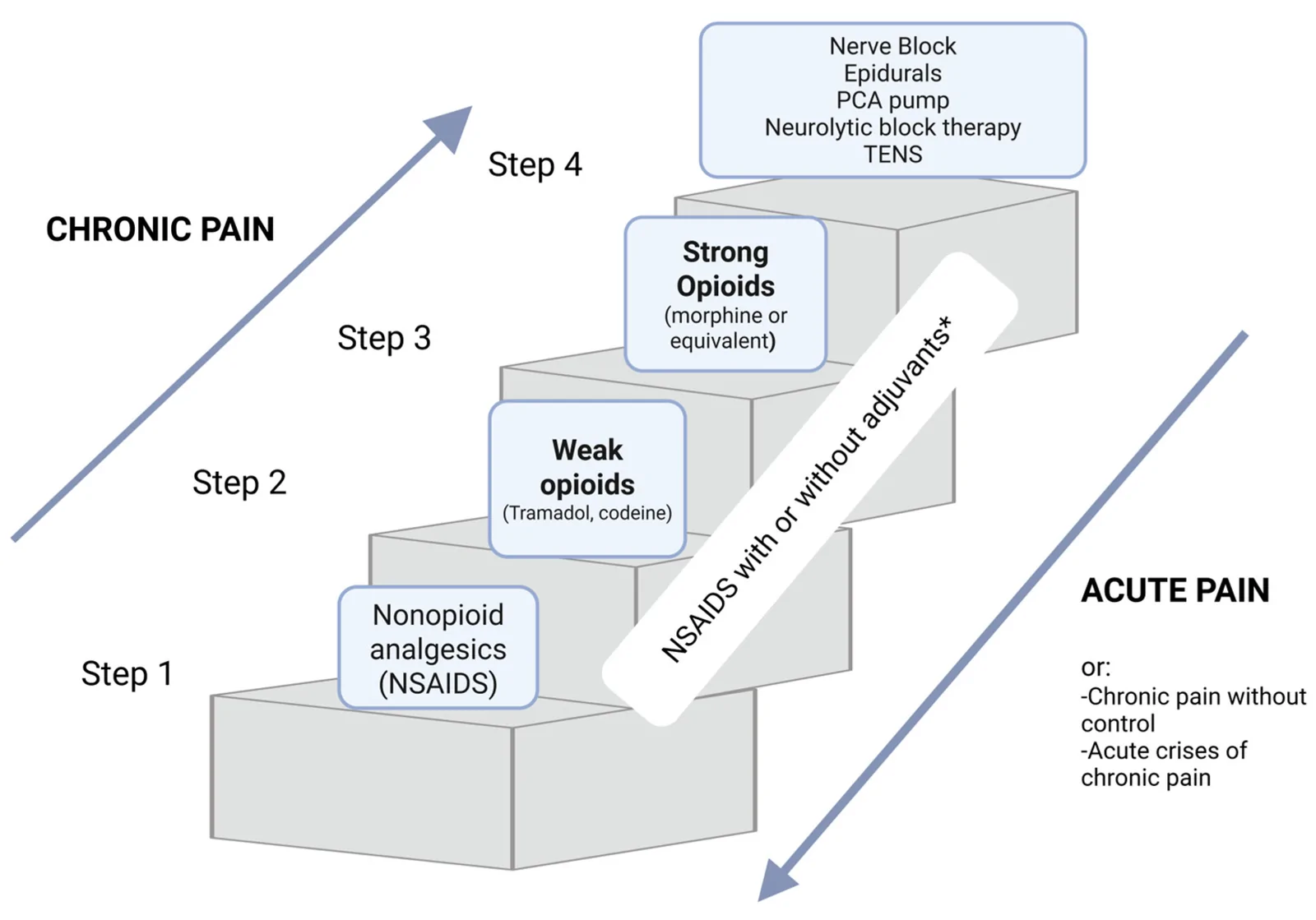
The WHO analgesic ladder is depicted as a four-step process. Each step represents escalating pain management, from non-opioid treatments (step 1) to stronger opioids and invasive techniques (step 4). Acute pain follows a “step-down” approach, starting with strong interventions and reducing intensity, while chronic pain follows a “step-up” approach. * Adjuvants, such as anticonvulsants and antidepressants, were integrated at all levels. Created in BioRender. Cordero, J. (2026) https://BioRender.com/t87a462, accessed on 5 February 2026.

Modern pain management tailors treatment to the severity and type of pain (Figure 3, Supplementary Table S2). Acute pain is addressed using a stepwise approach, escalating to strong opioids when necessary and de-escalating once controlled to minimize risk. For chronic pain, a multimodal strategy combines opioids with therapies such as nerve blocks to manage complex cases while reducing long-term dependence and ensuring effective, individualized care [2,29,30,31].

## 4. Clinical Use of Opioids in Acute, Chronic, and Cancer Pain

### 4.1. Opioid Use in Acute Pain

Acute pain, typically lasting less than one month, requires a tailored management approach, particularly in opioid-naïve patients. Non-opioid therapies, such as paracetamol, NSAIDs (ibuprofen, diclofenac, ketorolac), and inhaled analgesia (methoxyfluorane, nitrous oxide), should be the first-line treatment due to their effectiveness [32], although inhaled analgesics are not approved in certain countries, such as Spain.

Additionally, systematic reviews indicate that opioids provide no superior benefit over NSAIDs for musculoskeletal injuries [high certainty, conditional recommendation], including sprains, whiplash, muscle strains, and kidney stone-related pain [32]. Furthermore, in headache management, the American Headache Society discourages the use of opioids and butalbital for recurrent headaches due to dependency risks, with insufficient evidence supporting opioid use for treating episodic migraines [33].

However, opioids remain essential for severe acute pain or when non-opioid treatments are contraindicated or ineffective [high certainty, strong recommendation] [34,35,36]. It could include major traumatic injuries (e.g., crush injuries and burns) and invasive surgical procedures associated with moderate to severe postoperative pain unmanageable with NSAIDs [2,32,37,38].

Guidelines recommend a step-down approach using the reverse analgesic ladder [29,30,31], where treatment begins with strong opioids for severe pain and gradually transitions to weaker opioids and eventually non-opioid options as the pain subsides [29,30]. Immediate-release opioids are preferred for acute pain because they reduce overdose/sedation risk and allow safer dose control compared to long-acting formulations [39]. Additionally, a multimodal strategy combining continuous non-opioid analgesics with non-pharmacologic interventions is recommended for optimal pain management [29,30,40].

### 4.2. Management in Acute Pain

In moderate pain (NRS 4–6, VAS 40–60): if analgesia is insufficient with NSAID’s and metamizole (8–16 mg/kg orally or 1 g IV), further pain control is needed with minor opioids such as oral codeine (30–60 mg) or tramadol (50 mg) [2,38].

**Figure 4:**
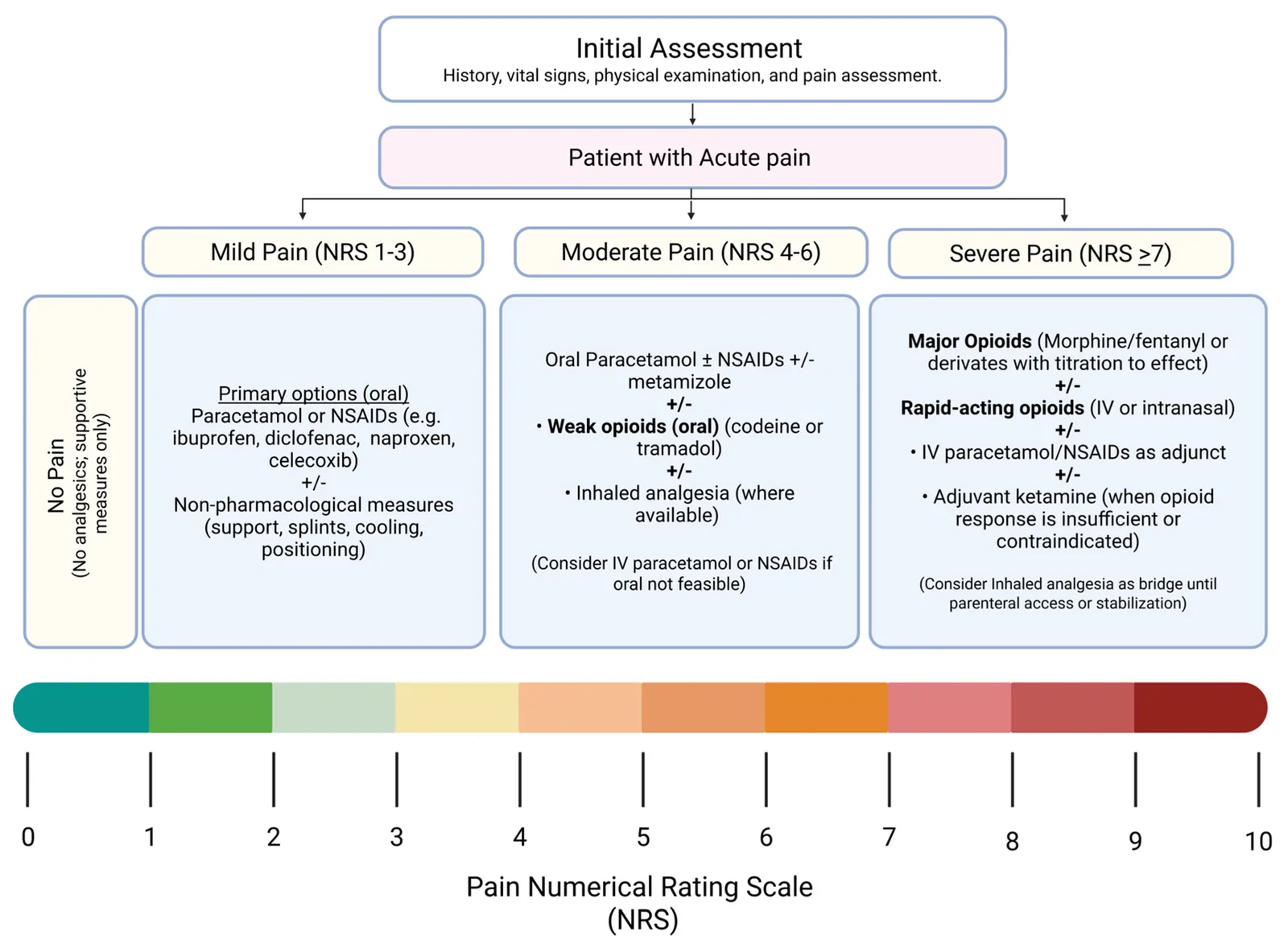
Algorithm for the management of acute pain. The initial assessment included evaluation of the pain type, location, and severity (mild, moderate, or severe). The Numerical Rating Scale (NRS) is a highly validated measure of pain intensity and is particularly useful for assessing clinically meaningful changes over time. A stepwise approach is followed, starting with non-pharmacological methods, followed by paracetamol/NSAIDs for mild pain and inhaled analgesics for moderate pain. The figure emphasizes the role of opioids in the treatment of acute pain. Intravenous opioids, such as morphine and fentanyl, are the primary choices for severe pain, requiring careful titration to minimize sedation and respiratory depression. Codeine and tramadol are options for treating moderate pain. Ketamine iv 0.1 mg/kg, repeated after 10 min, or inhaled 0.7 mg/kg with subsequent dosing of 0.3–0.5 mg/kg serves as third-line therapy when opioids are insufficient, with continuous reassessment for dose adjustment. The algorithm was synthesized from multiple clinical guidelines and peer-reviewed literature on acute pain management and summarized from sources [1,2,3,32,38,41,42,43,44,45,46]. Created in BioRender. Cordero, J. (2026) https://BioRender.com/t95h0o7, accessed on 5 February 2026.

In severe pain (NRS 7–10, VAS 70–100): Inhaled analgesics such as methoxyflurane or nitrous oxide (1 × 3 mL vial, maximum of 2 × 3 mL vials) are preferred until the patient can be transferred to an emergency setting or a stronger analgesic is established until IV opioids are applied as the primary choice, with IV morphine (2–3 mg titrated, with subsequent dosing at no less than 2 min intervals at 0.1 mg/kg) [41,42,43] or fentanyl (IV 0.05 mg or inhaled 50–100 μg, repeated at intervals of less than 10 min until the pain is controlled) being the drugs of choice [44,45]. If needed, adjuvant treatments include intravenous paracetamol (1 g) and minor opioids such as oral codeine (30–60 mg), tramadol (50 mg), or oxycodone (10 mg) [2]. The management of acute pain is shown in Figure 4.

If opioids are prescribed, clinicians must closely monitor patient responses, balancing pain relief with potential risks, such as sedation, respiratory depression, and dependency [39]. Intravenous opioids should not be used concurrently to prevent adverse effects, and naloxone should always be available in emergencies. Furthermore, opioid dosages must be carefully calculated, with regular follow-ups to assess effectiveness and minimize risks, especially when co-prescribed with benzodiazepines or other sedative medications, to prevent respiratory depression [32].

### 4.3. Management in Chronic Pain

The European Pain Federation emphasizes the proper use of opioids for chronic pain treatment, especially in areas with limited access to specialized pain clinics, where primary care providers are responsible for patient care to enhance functionality rather than completely eliminating discomfort. While crucial for severe pain management, opioids require ongoing evaluation of risks and benefits due to their potential side effects and addiction [5]. Dosage should be adjusted gradually according to pain type and intensity, and harmful effects on daily activities, psychological factors, and relevant health conditions, such as digestive, liver, kidney, or lung issues, should be avoided [35]. Nevertheless, prompt pain relief should not be delayed, even in the absence of a definitive diagnosis [37].

Reviewing patients’ controlled substance history through Prescription Drug Monitoring Programs (PDMPs) helps identify potential overdose risks, and addressing mental health conditions, such as depression and anxiety, is essential for optimizing pain treatment outcomes [8,46].

In management, if opioid therapy is initiated for acute, subacute, or chronic pain, clinicians should prescribe the lowest effective dose of immediate-release opioids rather than extended-release or long-acting formulations, with close follow-up to reassess benefits and harms shortly after starting or escalating therapy [30,32]. Several studies based on healthcare providers should assess the risks and benefits of opioid therapy within 1–4 weeks of treatment initiation or dosage increases [32,37], avoiding the combination of opioids with other central nervous system depressants, such as benzodiazepines, unless absolutely necessary and the benefits outweigh the potential risks [8,47].

Management should follow the WHO analgesic ladder (Figure 3), progressing to strong opioids when needed. For persistent pain, scheduled analgesic administration is recommended, with additional medications available for breakthrough pain, always adjusting the doses progressively and combining different drug classes, including gabapentinoids and antidepressants, if necessary, to reduce opioid requirements and minimize side effects [36].

### 4.4. Chronic Non-Cancer Pain

The use of opioids for managing chronic non-cancer pain (CNCP) is controversial because of limited long-term efficacy data, safety concerns, and the risk of addiction [Low certainty, Conditional recommendation] [32,35,48,49].

Despite established clinical guidelines, prescribing practices remain inconsistent, with opioids often prescribed for conditions like arthritis and headaches, where their benefit is uncertain or contraindicated by comorbid conditions such as COPD, demonstrating effectiveness only in short-term trials for neuropathic pain [Moderate certainty, conditional recommendation] [34,49,50,51]. Clinical guidelines recommend prioritizing non-opioid therapies, especially in patients with a history of substance use disorders or serious psychiatric conditions, reserving opioids for cases where pain persists despite optimized treatments [high certainty, strong recommendation]. In case it is necessary when the rest of the treatment lines fail, it is recommended to start with the lowest effective dose, which should not exceed 50–90 mg morphine equivalents daily, with adjustments based and monitoring therapeutic response and side effects [low certainty, conditional recommendation]. However, evidence supporting these practices remains weak and their implementation inconsistent [32,35].

### 4.5. Cancer Pain

#### 4.5.1. The Role of Weak Opioids in Cancer Pain Management

Weak opioids, such as tramadol and codeine, are commonly used for mild-to-moderate pain in Step 2 of the WHO analgesic ladder and are often combined with NSAIDs or acetaminophen to enhance pain relief [30,31]. However, their effectiveness in managing cancer pain is limited [low certainty, conditional recommendation], with over 50% of patients requiring a transition to strong opioids within two weeks due to inadequate pain control [37,46,52]. This raises questions about their necessity, as starting with low doses of strong opioids, such as morphine, oxycodone, or fentanyl, may provide faster and more reliable relief.

Additionally, genetic variations in opioid metabolism can cause unpredictable results, particularly in low- and middle-income countries [53], where weak opioids can be costly and access to stronger opioids is restricted [11].

Strong opioids, such as morphine, oxycodone, fentanyl, and hydromorphone, are recommended [high certainty, strong recommendation]. A Cochrane review of 152 randomized controlled trials (RCTs) involving 13,000 participants found that over 90% of patients achieved significant pain relief within 10–14 days using oral morphine or fentanyl patches. However, side effects such as constipation and nausea affect up to 77% of patients, requiring a change in therapy in 10–20% of patients. While no significant differences in pain relief exist among various strong opioids, side effects like CNS complications are more common with oral morphine compared to transdermal fentanyl [46].

The use of opioids is further complicated by tolerance, individual variability in metabolism, and the risk of misuse of these drugs. This underscores the importance of personalized opioid therapy tailored to tolerance, side effects, and pain type. Clinicians should employ tools such as the Opioid Risk Tool or CAGE questionnaire to assess misuse risk and ensure close monitoring, including follow-ups and urine drug testing in high-risk patients [54,55].

#### 4.5.2. Management of Moderate to Severe Cancer Pain

For mild to moderate pain, medications such as tramadol (50–100 mg every 4–6 h) or codeine (30–60 mg every 4 h) can be initiated. In cases of severe pain, stronger opioids such as morphine (5–10 mg every 4 h), oxycodone (5–10 mg every 4–6 h), or tapentadol (50–100 mg every 4–6 h) are recommended. Combining multiple opioids should be avoided, as should long-acting opioids such as fentanyl or methadone, due to their higher risk of adverse effects. However, tramadol, tapentadol, and oxycodone are particularly effective for pain with neuropathic components [56,57].

Morphine therapy should begin with immediate-release morphine (2.5–10 mg every 4 h), followed by adjustment to extended-release formulations based on total daily doses. Rescue doses should be one-sixth or one-tenth of the total daily doses. For example, if a patient takes 30 mg/day of extended-release morphine (15 mg every 12 h), the rescue dose would be 3–5 mg of immediate-release morphine. If pain is uncontrolled, a 30% dose increase may be necessary [32,46].

**Figure 5:**
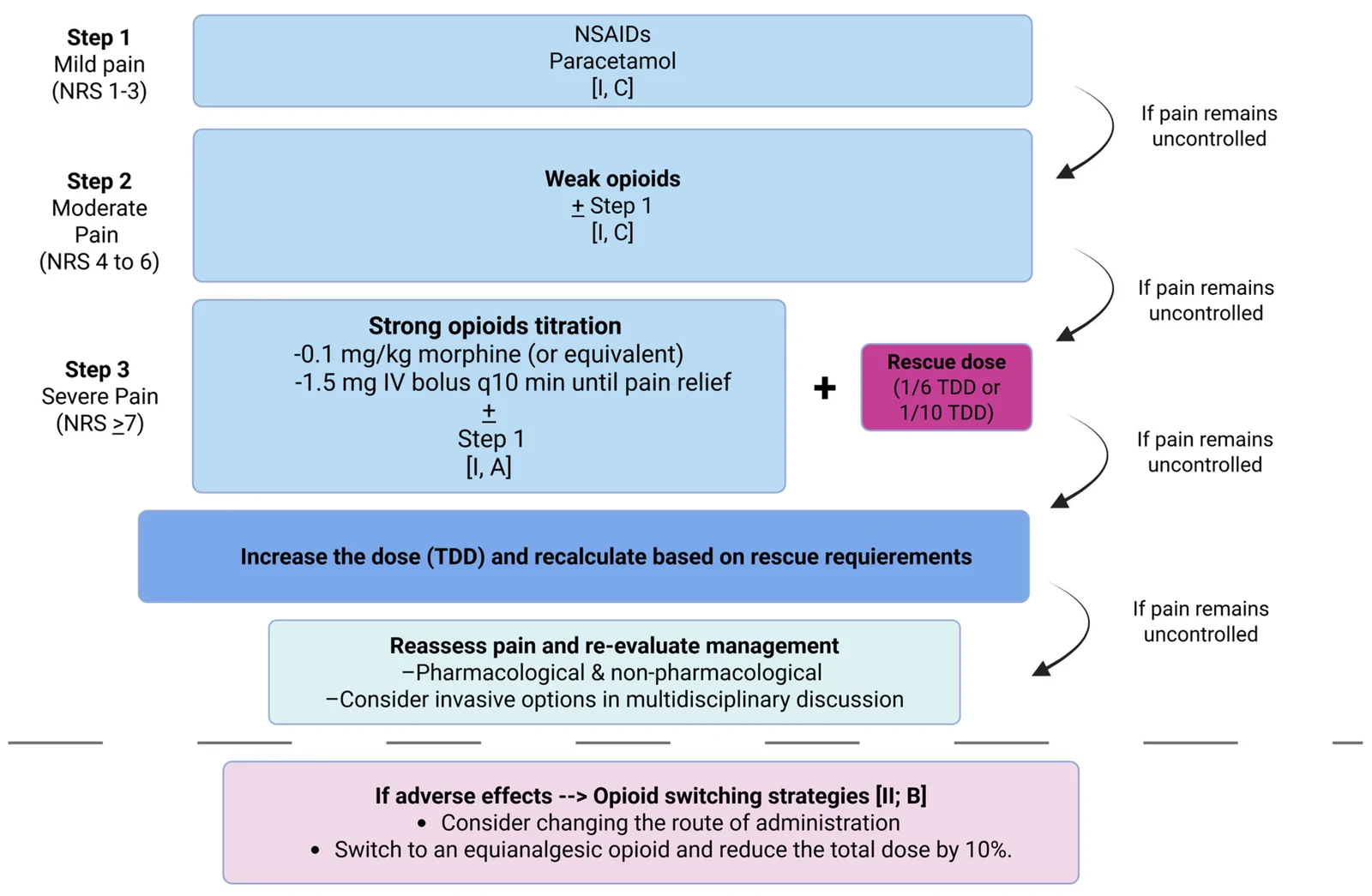
Algorithm for Chronic Cancer Pain Management. Chronic cancer pain management follows the WHO analgesic ladder, starting with non-opioids (paracetamol and NSAIDs) and progressing to weak and then strong opioids for effective relief. Morphine, oxycodone, and fentanyl are preferred for severe pain, with dose titration and management of side effects. Regular reassessment ensures safety, whereas scheduled and rescue dosing maintains continuous pain control. Authors’ synthesis of chronic cancer pain management based on the WHO Analgesic Ladder. The concepts were informed by the ESMO Clinical Practice Guidelines on cancer pain [37]. Created in BioRender. Cordero, J. (2026) https://BioRender.com/5c71zq7, accessed on 5 February 2026.

Treatment should be regularly monitored, with reassessments every 8–12 weeks to determine the need for continuation. For opioid withdrawal, a 10% reduction in the initial dose per week is recommended. If withdrawal symptoms occur, clonidine (0.1–0.2 mg orally every 6 h) can be used. In cases of opioid dependence, referral to specialized units for behavioral therapy and methadone treatment is recommended [56,57]. The management of opioids in chronic pain is shown in Figure 5.

For breakthrough cancer pain, short-acting rescue opioids are selected based on pain kinetics. Rapid-onset episodes are preferentially treated with transmucosal fentanyl formulations, initiated at the lowest dose and individually titrated, whereas immediate-release morphine or oxycodone at 10–15% of the total daily opioid dose is suitable for predictable or slower onset [58,59]. Among the fentanyl formulations, intranasal fentanyl provides the fastest absorption and shortest time to analgesia, sublingual fentanyl shows rapid but slightly delayed peak, and buccal fentanyl has more gradual absorption [59,60,61,62].

## 5. Opioid Use in Neuropathic Pain

Neuropathic pain arises from damage or dysfunction of the nervous system and often manifests as burning, shooting, or tingling sensations. Conditions such as diabetic neuropathy, postherpetic neuralgia, and trigeminal neuralgia are common causes of neuropathic pain. This type of pain can be particularly challenging to treat because conventional analgesics are often ineffective [63,64,65].

**Figure 6:**
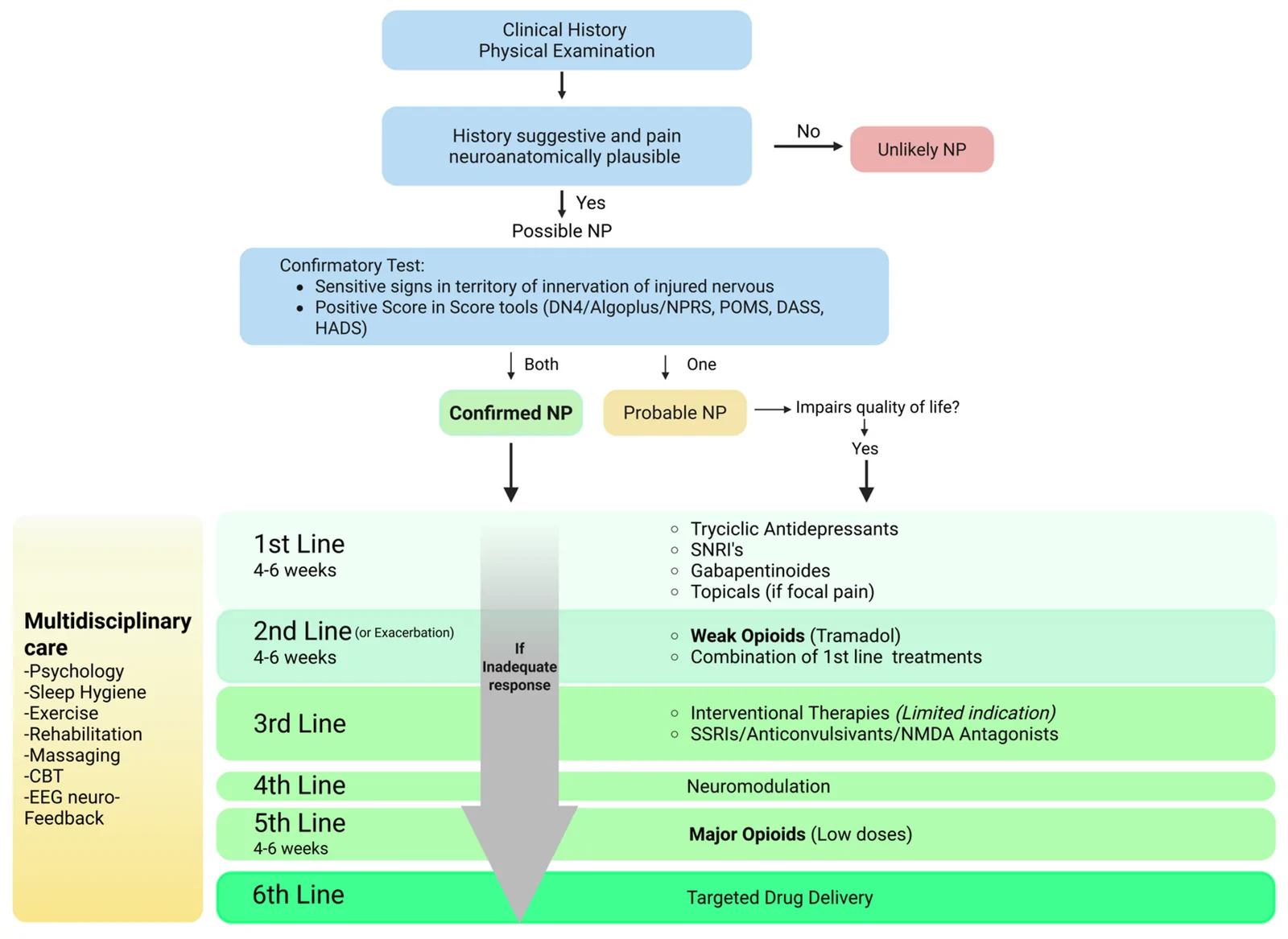
Algorithm for Neuropathic Pain Diagnosis and Management. It prioritizes non-opioid treatments due to the limited efficacy of opioids and the high risk of side effects. First-line options include TCAs (amitriptyline), SNRIs (duloxetine), and gabapentinoids (gabapentin, pregabalin). Opioids, such as tramadol, are second-line drugs but are reserved for refractory cases. Strong opioids (morphine, oxycodone, and methadone) are considered fourth-line drugs after neurostimulation. Their use is restricted to severe, persistent pain when other treatments fail, with doses carefully titrated to minimize the risks. Targeted drug delivery (TDD) using intrathecal opioids is the last resort. Conceptual model for neuropathic pain assessment and treatment synthesized from multiple clinical guidelines and reviews [22,36,64,70]. Created in BioRender. Cordero, FJ. (2026) https://BioRender.com/jf95tf4, accessed on 5 February 2026.

The role of opioids in the treatment of neuropathic pain is controversial. Opioids may provide some pain relief [Moderate certainty, Conditional recommendation], but they are generally considered less effective than other treatments, such as anticonvulsants (e.g., gabapentin and pregabalin) and certain antidepressants (e.g., amitriptyline, duloxetine) [50,51,66]. The adverse effects of opioids are also more pronounced in patients with neuropathic pain, with high rates of treatment discontinuation due to side effects. Given these issues, opioids are generally reserved for cases in which other therapies have failed and should be used with caution [64,67,68,69,70]. The algorithm for the diagnosis and management of neuropathic pain is presented in Figure 6.

Tramadol, an opioid with serotonin and norepinephrine reuptake inhibition, is effective for moderate-to-severe neuropathic pain at doses of 50–400 mg/day. However, its side effects (dizziness, nausea, dependence) limit its use to short-term cases or when first-line treatments (gabapentin, pregabalin, SNRIs, and TCAs) fail [35,69]. Combination therapy (e.g., gabapentin with opioids or pregabalin with TCAs) may enhance pain relief while minimizing individual drug doses, although it increases the risk of side effects. Due to the risk of polypharmacy, careful monitoring is essential, especially for long-term use [64,65,67,70,71].

Low-dose opioids are a fourth-line treatment for neuropathic pain [low certainty, conditional recommendation] and are considered only after neurostimulation has been attempted (per NICE guidelines). While short-term use (8 days to 8 weeks) of opioids such as oxycodone, morphine, methadone, and levorphanol provides moderate pain relief (NNT of 3.7 for morphine), their long-term efficacy is limited due to significant side effects. Guidelines recommend starting with immediate-release forms, keeping doses below 50 mg/day morphine equivalent, and avoiding doses above 90 mg/day unless strictly necessary [70].

## 6. Opioid Use in Other Pain Conditions and New Therapies

Compared with other pain management strategies, such as nonsteroidal anti-inflammatory drugs (NSAIDs) or tricyclic antidepressants, opioids have not consistently been shown to offer superior pain relief [35,36].

Opioids are generally not recommended for conditions such as fibromyalgia, chronic low back pain (CLBP), visceral pain, or chronic headaches due to their limited efficacy and potential for harm [12,72,73,74].

In fibromyalgia, which is classified as a nociplastic pain condition (Supplementary Table S2), strong opioids are generally avoided [very low certainty, strong recommendation against], as they may exacerbate symptoms through opioid-induced hyperalgesia, with tramadol providing only limited benefit. Non-opioid options, such as antidepressants (e.g., duloxetine) and anticonvulsants (e.g., pregabalin), are preferred [72,73,74].

For CLBP, while short-term opioid use may provide some relief, its impact on functionality is modest, and prolonged use carries a high risk of tolerance, dependence, and misuse. NSAIDs and physical rehabilitation are prioritized, with opioids reserved for short-term use as part of a multimodal approach [68,75,76].

In osteoarthritis, direct comparative evidence shows that opioids do not significantly outperform NSAIDs in pain relief while producing substantially more adverse effects. The SPACE trial demonstrated no superiority of opioids over NSAIDs for pain and function at 12 months in chronic knee and hip osteoarthritis, despite a higher incidence of drug-related harm [77]. These findings were further supported by a network meta-analysis showing that NSAIDs provide better pain control and functional improvement than opioids at the population level [78].

In visceral pain conditions, such as IBS or chronic pelvic pain, opioids are discouraged because of their limited efficacy and high risk of gastrointestinal side effects. Non-opioid treatments, including dietary changes and behavioral therapies, are preferred, with opioids considered only for short-term use in specific cases [79,80]. Similarly, opioids are not recommended for chronic headaches or facial pain because of the risk of medication overuse headache (MOH). Preventive treatments, such as beta-blockers, anticonvulsants, and triptans, are preferred for long-term management [33]. In osteoarthritis, for example, direct comparisons show that opioids do not significantly outperform NSAIDs in terms of pain relief, although they carry a much higher burden of adverse effects than NSAIDs [35,36].

Recent evidence suggests that nebulized opioids are an emerging off-label option for symptom control when oral or parenteral routes are not feasible. Survey data show the limited but growing clinical use of nebulized morphine and fentanyl in palliative care, mainly for pain, dyspnea, and cough, with high interindividual variability [81]. Pharmacokinetic studies indicate that modern aerosol devices may improve bioavailability and onset compared with conventional jet nebulizers, particularly for fentanyl [82]. However, controlled trials have shown inconsistent clinical benefits, underscoring the need for further evidence and careful patient selection [83,84].

### Most Recent Opioid Therapies: Tapentadol and Buprenorphine

Tapentadol, approved by the FDA in 2008, is a newer opioid with a dual mechanism of µ-opioid receptor agonism and noradrenaline reuptake inhibition, making it particularly suitable for neuropathic or mixed pain states, including cancer-related pain [85,86]. Clinical trials have shown that its analgesic efficacy is comparable to that of morphine and oxycodone [high certainty, strong recommendation], with a more favorable gastrointestinal and central nervous system tolerability profile, likely related to its reduced µ-opioid receptor load [63,69,70]. Nevertheless, toxicological and post-marketing data indicate persistent risks of misuse, serious adverse events, and rare fatalities, particularly in overdose or high-risk populations, necessitating careful patient selection and monitoring [87]. Structured tapering remains advisable, as evidence supporting optimal discontinuation strategies is still limited and largely based on expert opinions rather than robust comparative trials [88].

Buprenorphine, a high-affinity partial µ-opioid receptor agonist, represents an emerging alternative to traditional full µ-agonists and is increasingly used in chronic pain management, particularly in patients with renal impairment, advanced age, respiratory comorbidities, or elevated risk of opioid-related adverse events [89]. Its ceiling effect on respiratory depression, without loss of analgesic efficacy, may reduce the risk of overdose [Moderate certainty, Conditional recommendation] [90]. However, its strong receptor binding complicates opioid rotation, as initiation in the presence of full agonists may precipitate withdrawal symptoms. Structured micro-induction or micro-dosing strategies have shown promise in facilitating safer transitions, although the supporting evidence remains largely observational [91]. Buprenorphine’s role in cancer pain continues to expand, mainly as a second-line option during opioid rotation or in patients who develop opioid-induced adverse effects [46,92,93].

Given the variability observed across pain syndromes and contexts, we summarized the certainty of the evidence and strength of the recommendations supporting opioid use using the GRADE framework. Table 1 presents a cross-sectional synthesis of the main clinical questions addressed in this review.

## 7. Clinical Safety and Risk Management in Opioid Therapy

### 7.1. Risks, Adverse Effects, and Safety Considerations

The long-term efficacy and safety of opioids in chronic pain remain highly uncertain, with limited benefits and significant risks. While opioids provide short-term pain relief, studies have shown modest reductions in pain intensity but minimal improvement in functionality over time [30,36,103]. Side effects such as nausea, dizziness, and constipation, along with complications such as tolerance and opioid-induced hyperalgesia (OIH), often lead to discontinuation [35,104].

Discontinuation rates vary according to the administration method. Noble et al. reported that oral opioids have a 10.3% discontinuation rate due to inadequate pain control, while transdermal fentanyl shows slightly better outcomes with a 5.8% discontinuation rate. Intrathecal opioids present potential benefits but have inconsistent results, making their long-term efficacy unclear [35].

The risks of long-term opioid therapy include abuse, misuse, and dependence. Abuse, with higher doses (exceeding 200 mg morphine equivalents/day), significantly increases the risk of fatal overdose, particularly with synthetic opioids such as fentanyl [55,104,105,106,107]. Other serious complications include respiratory depression, hormonal imbalances, and cardiovascular issues, all of which can severely affect the quality of life [54,107,108].

Additionally, opioid-induced hyperalgesia (OIH), characterized by increased pain sensitivity, affects 24–39% of patients. Recent studies by Dhingra et al. showed that 48.5% of 489 patients undergoing methadone maintenance therapy (MMT) still experienced significant pain despite high opioid doses [54,92,100].

Psychiatric comorbidities, such as anxiety, depression, and PTSD, further exacerbate the risk of opioid misuse, making it crucial to stabilize these conditions before initiating opioid therapy. The public health impact is severe, particularly in North America, where opioid-related hospitalizations and deaths have increased [109,110]. In Ontario, for example, opioid-related treatment program admissions doubled between 2004 and 2013, reflecting a broader crisis of opioid misuse and dependence [93,105,107,111].

### 7.2. Opioid Rotation and Optimization of Therapy

Opioid rotation, which involves switching between opioids or routes of administration, is a common practice to improve pain control and reduce side effects, particularly in cases of opioid-induced hyperalgesia, tolerance, or poor side effect management [5].

A systematic review of opioid rotation strategies highlighted that while no single opioid is universally superior, switching opioids often improves pain management, although side effects are rarely fully mitigated [112].

One exception is methadone, a potent synthetic opioid with unique pharmacokinetic properties, which is particularly effective in cases where patients have developed tolerance to other opioids [36,37]. Methadone, a potent synthetic opioid with a unique dual action as an opioid receptor agonist and NMDA receptor antagonist, is especially effective in managing refractory pain and tolerance but requires careful dosing and monitoring due to its complex pharmacokinetics. It is typically reserved for high-dose opioid cases or refractory pain [92].

The opioid rotation table is presented in Supplementary Materials (as Supplementary Figure S2).

## 8. Opioid Abuse, Misuse and Dependence/Addiction in Clinical Practice

Because the literature employs numerous and inconsistently defined terms describing problematic opioid use, prior consensus efforts (IMMPACT and ACTTION) sought to standardize terminology and provide operational definitions to enable comparison across studies. On the basis of these efforts, three core constructs were established—misuse (use contrary to prescription), abuse (intentional non-medical use), and addiction (compulsive use despite harm)—although reported rates in chronic pain populations remain highly variable [40]. Nonetheless, up to eight related terms have been described in the literature (e.g., pseudodependence, dependence), and many studies refer broadly to “opioid dependence” without differentiating among these constructs, further complicating prevalence estimates.

From a pharmacological standpoint, physical dependence refers to a physiological adaptation to opioids such that abrupt cessation or antagonist administration precipitates withdrawal; importantly, it can occur during appropriate medical use and could not result in addiction. In contrast, addiction (or opioid use disorder) is defined as a chronic, relapsing condition characterized by compulsive opioid use despite harm, loss of control over use, craving, and continued consumption despite negative consequences [113]. In the DSM-5, these behavioral features are captured under the definition “opioid use disorder”, and tolerance or withdrawal alone are insufficient for diagnosis, as both may accompany addiction but are neither necessary nor sufficient [114]. Despite this differentiation, the interchangeable use of terms has hindered classification and interpretation. As noted by Fishbain et al., among 24 reviewed studies, only 7 applied acceptable diagnostic criteria for substance-use disorders, and only 3 explicitly evaluated psychological dependence and compulsive use—the defining features of addiction—with reported rates ranging from 3% to 16% [115].

Collectively, nonstandardized terminology and overlapping constructs have limited reliable estimates of problematic opioid use. Because most studies treat dependence and addiction as equivalent, we merged these categories for epidemiological reporting.

Throughout this manuscript, epidemiological prevalence estimates are reported using the aggregated term “dependence/addiction,” consistent with the terminology employed in the majority of studies, which do not distinguish between physical dependence and addiction when reporting rates. By contrast, within our conceptual discussion, “dependence” is reserved for physiological adaptation to opioids, whereas “addiction” is used to designate behavioral compulsivity and continued use despite harm.

### 8.1. Opioid Misuse and Abuse

Misuse was the most commonly reported issue, with prevalence estimates ranging from as low as 0.05% to as high as 81%, reflecting inconsistencies in definitions and methodological approaches [54].

Opioid abuse, characterized as intentional non-medical use for euphoria or other psychoactive effects, was examined in only one study, which reported a prevalence of 8% [116]. European pharmacovigilance data provide a quantitative real-world perspective on opioid misuse and abuse. Although Europe has not experienced an epidemic comparable to North America—where prescription opioids affected up to 1.7% of the population—medical opioid use has increased steadily, prompting safety concerns [117]. Analyses of the EudraVigilance database identified over 16,000 opioid-related adverse drug reaction reports over the last decade particularly for fentanyl, oxycodone and tramadol [118]. In national analyses, opioid abuse-related frequently involving polypharmacy and psychiatric comorbidity [119]. Additionally, disproportionality analyses using European pharmacovigilance data revealed significant reporting signals linking opioid exposure to neurocognitive adverse outcomes, including learning disorders, compared with non-opioid analgesics [120]. Collectively, these data indicate that while overall harm remains lower than in the U.S., misuse and long-term safety signals are measurable in Europe, underscoring the need for continued surveillance and balanced prescribing.

### 8.2. Opioid Dependence and Addiction

Opioid dependence is a multifactorial issue influenced by clinical practices, dosage, treatment duration, and socioeconomic factors [108,121]. Recent reviews indicate that, despite this heterogeneity, dependence rates tend to be lower than previously expected [23,122].

Estimates of opioid dependence varied significantly, from 0.03% to 34.1% [40]. Notably, studies have reported low dependence rates (0.03–11%) among patients without prior substance use histories [103,123,124]. Such marked discrepancies in prevalence estimates are most plausibly driven by differences in methodological approach, sampled populations, and the definitional frameworks applied to identify problematic opioid use.

Risk increases with high opioid dosages, long-term use, and concurrent use of substances like benzodiazepines [121,125]. Vulnerable populations include adolescents, older adults, individuals with mental health disorders, and those with a history of substance use. Furthermore, certain opioids like hydrocodone have been shown to possess a higher abuse potential compared to alternatives such as tramadol [123] and benzodiazepine co-use is strongly associated with an elevated risk of overdose [121].

### 8.3. Underlying Causes of Opioid Dependence: Interracial, Social, and Individual Factors

Opioid dependence is influenced by genetic, social, and individual factors. A key contributor to the variability in opioid response is genetic variation among racial and ethnic groups, which affects drug pharmacokinetics, pharmacodynamics, and susceptibility to dependence and overdose.

Genetic differences across racial and ethnic groups in genes involved in opioid pharmacokinetics and pharmacodynamics can substantially influence susceptibility to dependence, overdose, and variable therapeutic response. Relevant genetic variation has been described in pathways related to drug transport, metabolism, and opioid receptor function [126,127,128]. Moreover, genome-wide association studies have identified regulatory variants on chromosome 20 (near PCMTD2 and OPRL1) associated with pain intensity, suggesting additional genetic contributions to opioid responsiveness [129].

In addition to biological factors, racial disparities play a significant role in opioid dependence and its treatment. Multiple studies have documented that Black patients, for instance, receive up to 36% fewer opioids than White patients (5190 vs. 8082 MME) for comparable clinical conditions, a disparity observed across 91% of U.S. healthcare systems [7,130,131]. Importantly, evidence indicates that lower opioid prescription among Black patients reflects systemic bias rather than reduced analgesic need and is associated with inadequate pain control and worse patient-reported outcomes, particularly in acute and cancer-related pain settings [53,130].

Substantial geographic variations in opioid-related outcomes reflect broader socioeconomic inequalities and structural differences between health systems. Marked differences exist between the United States and Europe in opioid-related harm, largely reflecting the divergent prescribing practices and regulatory frameworks. In the U.S., prescription opioids have affected up to 1.7% of the population, contributing to substantially higher overdose mortality, whereas most European countries report lower prevalence and mortality rates despite increasing medical opioid use [11]. Nevertheless, European pharmacovigilance data show measurable safety signals, with opioid-related adverse drug reactions accounting for approximately 10–15% of national opioid safety reports, frequently involving fentanyl, oxycodone, and tramadol [132,133]. Global policy analyses emphasize that these data support the need for balanced regulatory approaches that limit non-medical use while ensuring adequate access to essential analgesics, particularly within universal health coverage systems.

### 8.4. Individual Risk Factors for Opioid Addiction

Certain groups, such as adolescents, older adults, individuals with mental illness, and those with a history of substance use, are at a higher risk for opioid dependence due to biological and psychosocial vulnerabilities [134]. Key risk factors include high doses (≥100 MME), long-term use (≥3 months), and concurrent benzodiazepine use [32,135].

The TROUP study reported a 2.9–3.2% rate of post-treatment opioid addiction, with younger adults facing up to 11.4 times greater risk than older adults [40,107,121]. Hydrocodone showed higher abuse potential (4.4–5.5%) compared to NSAIDs and tramadol [40].

Children of parents with opioid addiction are at an elevated risk for behavioral disorders and family disruption—only 18% of these families remain intact versus 88% in the general population [136]. Low socioeconomic status and parental substance use further compound the risk of psychosocial dysfunction [101,137].

### 8.5. Treatments for Opioid’s Dependence

For individuals with opioid dependence, especially in non-cancer pain, medication-assisted therapies (MATs), including methadone and buprenorphine, are highly effective. These treatments help reduce cravings, stabilize brain chemistry, and significantly lower the risk of overdose in patients with OUD. Despite the lingering stigma that portrays them as drug substitutes, extensive evidence supports their role in recovery and in reducing opioid-related mortality [94,138]. Beyond maintenance therapy, emerging evidence supports structured, patient-centered opioid de-escalation strategies that integrate pharmacological management with psychological and educational support. A clinical trial has demonstrated that combining education and skill-based interventions with medical care substantially improves outcomes. After 12 months, 29% of participants in the intervention group successfully discontinued opioid use compared to 7% in the usual care group (odds ratio: 5.55, 95% CI: 2.80–10.99). These sessions focused on self-management strategies and support for opioid tapering [95,102].

Contemporary trials further indicate that multidisciplinary programs incorporating education, behavioral support, and longitudinal follow-up can facilitate meaningful opioid dose reduction or discontinuation without worsening pain-related function or quality of life [95,96]. Nevertheless, pragmatic studies suggest that more intensive psychological interventions do not consistently outperform simpler individualized tapering approaches grounded in shared decision-making, with both strategies achieving modest yet clinically acceptable reductions while preserving functional outcomes [97,98]. In patients with coexisting opioid use disorder, maintenance treatment with buprenorphine combined with behavioral therapy appears more feasible and functionally beneficial than tapering alone [139]. Notably, although opioid exposure may be substantially reduced, improvements in pain-related interference are not always observed, highlighting the need for complementary strategies to address pain and functional recovery, alongside dependence management.

**Figure 7:**
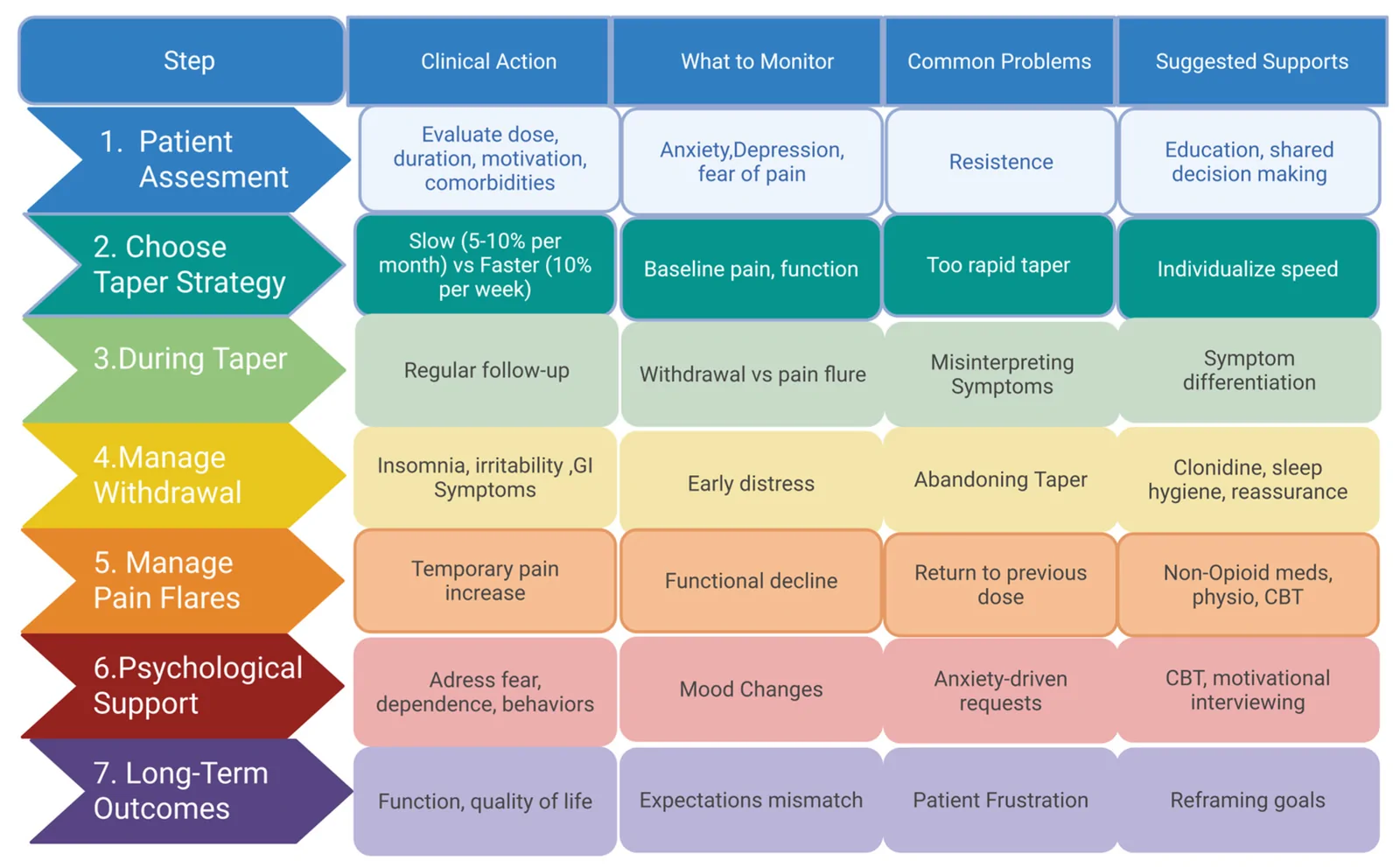
Practical considerations for opioid tapering in chronic pain (structured, patient-centered approach). This schematic summarizes a pragmatic tapering approach for clinical practice. Taper rate should be individualized according to baseline dose, duration of opioid therapy, comorbidities, and patient preferences. If clinically significant withdrawal symptoms occur, consider pausing the taper and resuming at a slower rate; symptomatic management (e.g., clonidine for autonomic symptoms) may be used in selected patients. Withdrawal symptoms typically include autonomic/affective features, whereas pain flares are more often localized and activity-related. Consider buprenorphine transition when appropriate. This figure is intended as an implementation aid and does not replace clinical judgment or local guidelines. Created in BioRender. Cordero, J. (2026) https://BioRender.com/20b3eep, accessed on 5 February 2026.

In clinical practice, opioid tapering requires a structured but flexible approach tailored to the individual patient. Key steps include careful initial assessment, shared decision-making, gradual dose reduction, and close monitoring to distinguish withdrawal symptoms from pain exacerbations. Commonly used approaches include a slow taper (e.g., ~5–10% dose reduction per month) for long-term or high-dose therapy and a faster taper (e.g., ~10% per week) when clinically appropriate. If clinically significant withdrawal symptoms occur, the taper can be paused and resumed at a slower rate, or minor temporary adjustments may be considered while maintaining close follow-up. Withdrawal typically presents with autonomic and affective symptoms (e.g., restlessness, sweating, insomnia, irritability), whereas pain flares are more often localized and activity-related. Supportive measures such as non-opioid analgesics, physical therapy, and psychological interventions may improve tolerability and adherence to the tapering process; symptomatic pharmacological options (e.g., clonidine for autonomic symptoms) can also be used in selected patients. In selected cases, transition to buprenorphine can represent a safer alternative during dose reduction. Importantly, long-term outcomes after discontinuation are often more favorable than expected, with many patients experiencing stable pain control and improved function once tapering is completed (Figure 7).

### 8.6. Practical Limitations

Although opioid prescribing guidelines and multimodal analgesic frameworks are evidence-based, their implementation in routine practice is frequently constrained by real-world organizational and structural barriers. Time pressure, limited resources, and fragmented interdisciplinary collaboration hinder comprehensive pain assessment and ongoing treatment reassessment [140]. At the system level, international analyses highlight persistent shortages in workforce capacity, infrastructure, and service availability, particularly outside high-income settings, limiting access to both opioid analgesia and essential non-pharmacological pain interventions [11,99]. In addition, considerable heterogeneity across study designs, populations, and diagnostic criteria complicates the interpretation of real-world opioid misuse and dependence rates, making it difficult to generate reliable population-level estimates. These challenges are compounded by deficiencies in pain education, with European surveys demonstrating significant knowledge gaps and misconceptions regarding opioid use among non-specialists, reflecting fragmented and insufficient undergraduate and postgraduate training [141,142]. From the patient perspective, poor continuity of care and restricted access to specialist follow-up after discharge further undermine the effective translation of evidence-based recommendations into sustained clinical benefits [143].

## 9. Conclusions

The role of opioids in pain management remains a complex intersection of medical necessity and significant public health concern. While opioids are indispensable for managing pain in several conditions (especially severe pain and chronic oncologic pain management), their use is controversial because of dependency risks and limited long-term efficacy. The wide variability in the reported rates of opioid misuse and dependence reflects substantial heterogeneity in definitions, study designs, and patient populations, as well as the overall low quality of available evidence. Consequently, the current data do not allow for reliable estimates of dependence risk. For clinicians, this underscores the importance of individualized risk assessment and personalized multimodal pain management strategies.

Global disparities in opioid access highlight the need for equitable healthcare policy. In addition, social conditions (such as substance abuse, psychiatric disorders, and low socio-cultural status), race, opioid metabolism variability, and unequal access are key drivers of dependence. Addressing these factors through social intervention is essential to ensure safer and more equitable pain management.

Furthermore, adverse dose effects and opioid-induced hyperalgesia complicate clinical management, necessitating tailored treatment plans, such as emerging therapies and opioid rotation strategies, which have improved pain control in complex cases. However, advancements in opioid pharmacology, including novel agents such as tapentadol and buprenorphine, offer promising alternatives with potentially fewer side effects.

Finally, integrating non-pharmacological therapies, such as cognitive–behavioral therapy, physical rehabilitation, and educational interventions, is crucial for reducing opioid dependence and enhancing patient outcomes. This should be accompanied by alternative pain therapies. Future pain treatment guidelines should include opioids, which have long been used effectively when managed with proper controls to prevent overdose, misuse and addiction. Although there are risks, they are generally lower in patients with pain, especially those without psychiatric or addiction issues. Additionally, new types of pain medications with mechanisms different those from of opioids show promise.

## Figures and Tables

**Table 1:** Summary of Evidence and Recommendations for Opioid Use in Pain Conditions (GRADE Framework).

Clinical Question/Claim	References	Study Design	Certainty (GRADE)	Strength of Recommendation
**Acute Pain**				
Weak opioids (codeine/tramadol) for acute pain	[2,32]	Clinical guidelines + RCTs	Low–Very Low	Conditional against (reserve for selected cases)
Opioids for severe traumatic or postoperative pain	[38]	Clinical guidelines + RCTs	High	Strong
Opioids for severe pain when non-opioids are contraindicated or ineffective	[2,38,42,44]	Clinical guidelines + RCTs	High	Strong
Opioids vs NSAIDs for acute musculoskeletal pain	[2,32]	Clinical guidelines + RCTs	High	Conditional for NSAIDs as first-line
Opioids for acute migraine	[33]	Guidelines + observational	Very Low	Strong recommendation against opioids
**Cancer Pain**				
Weak opioids for cancer pain	[37,46]	Guidelines + observational	Low	Conditional
Strong opioids for cancer pain	[32,46]	Clinical guidelines + RCTs + Systematic Reviews	High	Strong (first-line for moderate–severe cancer pain)
Tapentadol for neuropathic or mixed pain	[48,85,86,87,88]	Phase III RCTs + mechanistic + guidelines	High	Strong for neuropathic/mixed pain
				Conditional for cancer pain
Rapid-Onset Transmucosal Fentanyl (ROOs) for Breakthrough cancer pain	[59,61,62]	Observational/Narrative review/Delphi consensus	Moderate	Weak/Conditional
**Chronic Non-Cancer Pain (CNCP)**				
Opioids for CNCP (General)	[32,34,35,36,48]	Short-term RCTs + observational	Low	Conditional/Weak against
Opioids for chronic low back pain (CLBP)	[35,36,76]	Systematic review + clinical guidelines	Low	Conditional (short-term only)
Opioids vs. NSAIDs for osteoarthritis pain	[77,78]	RCT + network meta-analysis + guidelines	High	Strong against opioids as first-line
Opioids for neuropathic pain	[36,65,66,67]	Systematic review + RCTs (short duration)	Moderate (short-term)/Very Low (long-term)	Conditional/Weak against
Opioids for nociplastic pain (fibromyalgia/some CLBP)	[36,72,73,74,75]	Guidelines + observational + RCT	Very Low	Strong recommendation against
Opioids in visceral pain (IBD/IBS/CPP)	[79,80]	Cohort + clinical guidelines	Very Low	Strong recommendation against
**High-Risk Populations/Safety Strategies**				
Buprenorphine for chronic pain and high-risk patients	[37,46,89,90,91]	RCTs + observational cohorts + expert consensus	Moderate	Conditional (especially in renal impairment, advanced age, respiratory disease, high AE risk)
Opioid tapering strategies	[32,94,95,96,97]	Pragmatic RCTs + guidelines	Moderate	Conditional (individualized tapering)
				Strong against forced/rapid tapering
Multimodal strategies combining opioids + non-pharmacological therapies	[29,30,40,96,97,98,99]	Clinical guidelines + pragmatic RCTs	Moderate	Strong
Risk management (PDMPs, screening, follow-up)	[8,32,46,54,55]	Observational + quasi-experimental + consensus	Low–Moderate	Strong
Opioid Use Disorder/Dependence				
Buprenorphine/MAT for opioid dependence (OUD)	[89,100,101,102]	RCTs + cohorts + population-level + guidelines	High (mortality/overdose reduction)	Strong
Emerging/Investigational Uses				
Nebulized opioids (palliative/respiratory)	[81,82,83,84]	Observational + PK + Phase I–II trials	Very Low	Conditional/Investigational

Abbreviations: RCT, randomized controlled trial; SR, systematic review; MA, meta-analysis; NMA, network meta-analysis; Obs, observational study; PK, pharmacokinetics; Guideline, clinical practice guideline; Consensus, expert consensus; CNCP, chronic non-cancer pain; CLBP, chronic low back pain; NSAID, non-steroidal anti-inflammatory drug; IBD, inflammatory bowel disease; IBS, irritable bowel syndrome; CPP, chronic pelvic pain; OUD, opioid use disorder; MAT, medication-assisted treatment; PDMP, prescription drug monitoring program; AE, adverse event. GRADE classifications: Certainty of evidence reflects the overall confidence in the effect estimate and was categorized as high, moderate, low, or very low. The strength of recommendation was rated as strong, conditional/weak, or against, based on the balance between benefits and harms in clinical practice with color coding in the table: green indicates a strong positive association; yellow: weak/conditional positive association; orange: weak/conditional negative association; red: strong negative association; and blue indicates that the topic is currently under investigation. Grading was performed at the level of clinical questions rather than individual studies, consistent with the review’s narrative nature.

## Data Availability

No new data were created or analyzed in this study. Data sharing is not applicable to this article.

## Supplementary Materials

The following supporting information can be downloaded at: https://www.mdpi.com/article/10.3390/healthcare14040457/s1, Table S1: Detailed search strategies (MeSH/Emtree terms and free-text keywords) used across databases for the narrative literature review; Table S2: Overview of opioid ligands based on their receptor selectivity and pharmacological classification; Table S3: Types of pain; Figure S1: Study-selection flow diagram of the narrative literature review process; Figure S2: Opioid Conversion Chart.

## Abbreviations

The following abbreviations are used in this manuscript:

IASP	International Association for the Study of Pain
WHO	World Health Organization
NSAIDs	Non-Steroidal Anti-Inflammatory Drugs
CNCP	Chronic Non-Cancer Pain
CLBP	Chronic Low Back Pain
NRS	Numeric Rating Scale
VAS	Visual Analogue Scale
PDMPs	Prescription Drug Monitoring Programs
RCTs	Randomized Controlled Trials
CNS	Central Nervous System
GI	Gastrointestinal
OIH	Opioid-Induced Hyperalgesia
OUD	Opioid Dependence
MME	Morphine Milligram Equivalents
GPCRs	G Protein-Coupled Receptors
MOPR	Mu Opioid Receptor
DOPR	Delta Opioid Receptor
KOPR	Kappa Opioid Receptor
NOPR	Nociceptin Opioid Receptor
DRG	Dorsal Root Ganglion
NMDA	N-Methyl-D-Aspartate
TCAs	Tricyclic Antidepressants
SNRIs	Serotonin–Norepinephrine Reuptake Inhibitors
SSRIs	Selective Serotonin Reuptake Inhibitors
TDD	Targeted Drug Delivery
MMT	Methadone Maintenance Therapy
PTSD	Post-Traumatic Stress Disorder
IBS	Irritable Bowel Syndrome
MOH	Medication Overuse Headache
PAG	Periaqueductal Gray
RVM	Rostroventral Medulla
COPD	Chronic Obstructive Pulmonary Disease
FDA	Food and Drug Administration
ESMO	European Society for Medical Oncology
NICE	National Institute for Health and Care Excellence
