# Supplementary material for: Optimizing Opioid Use in Pain Management: A Comprehensive Review of Clinical Benefits, Risks, and Dependence
Source: Healthcare (Basel). 2026 Feb 11;14(4):457. doi: 10.3390/healthcare14040457 (PMC12940829; doi:10.3390/healthcare14040457)
Supplement: Supplementary file 1 [file healthcare-14-00457-s001.zip › healthcare-4078888-supplementary.pdf]

**Supplementary Materials**

## TABLE OF CONTENT


**Supplementary Tables**
**Supplementary Table S1.** Detailed search strategies (MeSH/Emtree terms and free-text keywords) used across databases for the narrative literature review.
**Supplementary Table S2.** Overview of opioid ligands based on their receptor selectivity and pharmacological classification
**Supplementary Table S3.** Types of Pain.

**Supplementary Figures.**
**Supplementary Figure S1.** Study-selection flow diagram of the narrative literature review process.
**Supplementary Figure S2.** Opioid Conversion Chart.

**Supplementary Table S1. Detailed search strategies (MeSH/Emtree terms and free-text keywords) used across databases for the narrative literature review.**

| Database | Search string (MeSH/Emtree terms + free text + Boolean operators) |
|---|---|
| **MEDLINE (PubMed)** | ((("Pain"[Mesh] OR "Acute Pain"[Mesh] OR "Chronic Pain"[Mesh] OR "Neoplasms/complications"[Mesh] OR "Palliative Care"[Mesh]) OR ("acute pain" OR "postoperative pain" OR "chronic pain" OR "noncancer pain" OR "chronic non-cancer pain" OR "cancer pain" OR "palliative care")) AND (("Analgesics, Opioid"[Mesh] OR "Opioid-Related Disorders"[Mesh]) OR (opioid* OR opiate* OR "opioid analgesics" OR morphine OR oxycodone OR fentanyl OR hydromorphone OR buprenorphine OR tapentadol OR tramadol)) AND (effectiv* OR analgesia OR function* OR "adverse event*" OR safety OR tolerabil* OR overdose OR "opioid-induced hyperalgesia" OR hyperalgesia OR misuse OR abuse OR "opioid use disorder" OR dependence OR addiction OR discontinuation OR taper OR withdrawal)) |
| **Embase** | (('pain'/exp OR 'acute pain'/exp OR 'chronic pain'/exp OR 'cancer pain'/exp OR 'palliative therapy'/exp) OR ("acute pain" OR "chronic pain" OR "non cancer pain" OR "cancer pain" OR "palliative care")) AND (('opioid analgesic agent'/exp OR 'opioid dependence'/exp) OR (opioid* OR opiate* OR morphine OR oxycodone OR fentanyl OR hydromorphone OR buprenorphine OR tapentadol OR tramadol)) AND (effectiv* OR analgesia OR function* OR adverse* OR safety OR overdose OR hyperalgesia OR misuse OR abuse OR dependence OR addiction OR discontinu* OR taper* OR withdrawal) |
| **Cochrane Library** | (MeSH descriptor: [Pain] explode all trees) AND (MeSH descriptor: [Analgesics, Opioid] explode all trees) AND ("pain" AND (opioid* OR morphine OR oxycodone OR fentanyl OR buprenorphine OR tapentadol OR tramadol) AND (chronic OR acute OR cancer) AND (review OR trial OR safety OR misuse OR dependence OR taper OR discontinu*)) |
| **Scopus** | ((acute OR chronic OR "non-cancer" OR cancer OR palliative) W/3 pain) AND (opioid* OR opiate* OR morphine OR oxycodone OR fentanyl OR hydromorphone OR buprenorphine OR tapentadol OR tramadol) AND (effectiv* OR function* OR safety OR "adverse event*" OR overdose OR |

| Database | Search string (MeSH/Emtree terms + free text + Boolean operators) |
|---|---|
| | hyperalgesia OR misuse OR abuse OR dependence OR addiction OR taper* OR discontinu* OR withdrawal)) |
| **ClinicalTrials.gov** | Condition or disease: pain; Other terms: (tapentadol OR buprenorphine) AND (chronic OR neuropathic OR cancer); Status: completed; Results: all |

**Supplementary Table S2. Types of Pain**. Pain is classified by duration (acute, chronic, episodic), mechanism (nociceptive, neuropathic, mixed, nociplastic), and etiology to guide treatment. Neuropathic pain involves sensory changes, while nociplastic pain stems from altered CNS processing. Emotional pain affects mental health. Genetic, environmental, and psychosocial factor

| | | |
|---|---|---|
| By duration | Acute (<6 months) | |
| | Chronic (>6 months) | |
| | Episodic (intermittent) | |
| Mechanism | Nociceptive (tissue damage) | Somatic pain |
| | | Visceral pain |
| | Neuropathic (nerve damage) | |
| | Nociplastic (altered central nervous system processing without tissue damage). E.g: Fibromyalgia | |
| | Emotional | |
| | Mixed | |
| Location | Headache, low-back pain, | |
| Etiology | Malignant, non-malignant | |
| Intensity | Mild | |
| | Moderate | |
| | Severe | |

**Supplementary Table S3. Overview of opioid ligands based on their receptor selectivity and pharmacological classification**. The table categorizes the compounds into two main groups: agonists (highlighted in green) and antagonists (highlighted in red). Each ligand is evaluated for its activity at the three major opioid receptor subtypes: mu (μ), delta (δ), and kappa (κ). Agonist compounds, such as Etorphine, Fentanyl, and DAMGO, exhibit strong or partial activation (+++ or P) of one or more receptors. These agents are typically used for analgesic effects and are characterized by their affinity profiles across receptor types. Conversely, antagonist compounds like Naloxone, Naltrexone, and CTOP display receptor-blocking properties (--- or –), preventing opioid receptor activation and are commonly used in the treatment of opioid overdose or dependence.

| **Opioid Ligands (Agonists)** | **μ** | **δ** | **κ** | |
|---|---|---|---|---|
| Etorphine | +++ | +++ | +++ | Agonist |
| Fentanyl | +++ | | | Agonist |
| Hydromorphone | +++ | | + | Agonist |
| Levorphanol | +++ | | | Agonist |
| Methadone | +++ | | | Agonist |
| Morphine | +++ | | + | Agonist |
| Sufentanil | +++ | + | + | Agonist |
| DAMGO (D-Ala[2], MePhe[4], Gly(ol)[5] enkephalin) | +++ | | | Agonist |
| Bremazocine | + | + | +++ | Agonist |
| Buprenorphine | P | | -- | Agonist |
| Butorphanol | P | | +++ | Agonist |
| Nalbuphine | -- | | ++ | Agonist |
| DPDPE ([D-Pen[2],[5]]-Enkephalin) | +++ | | | Agonist |
| U50,488 | - | ++ | | Agonist |
| Naloxone | – – – | – | – – – | Antagonist |
| Naltrexone | – – – | – | – – – | Antagonist |
| CTOP | – – – | | | Antagonist |
| Diprenorphine | – – – | – – | – – – | Antagonist |
| β-Funaltrexamine | – – – | – | ++ | Antagonist |
| Naloxonazine | – – – | – | – | Antagonist |
| nor-Binaltorphimine (nor-BNI) | – | – | – – | Antagonist |
| Naltrindole | – | – – – | – | Antagonist |
| Naloxone benzoylhydrazone | – – – | – | – | Antagonist |

**Supplementary Figure S1. Study-selection flow diagram of the narrative literature review process.**

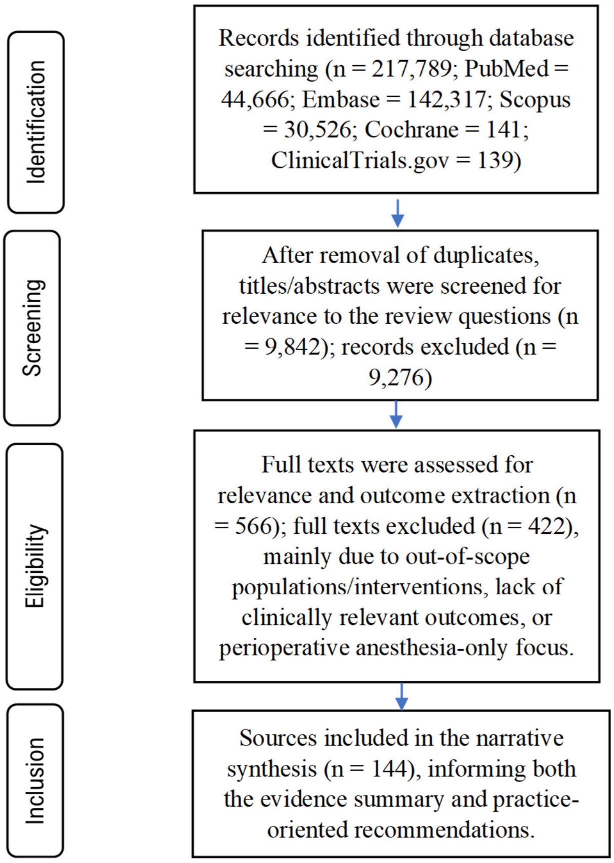

**Supplementary Figure S2.** Opioid Conversion Chart. Concept adapted from Wessex Palliative Physicians Handbook of Palliative Care, 9th Edition, 2019.

| Strong Opioids | | | | | | | | | | | | | | Patches | | Weak Opioids | |
|---|---|---|---|---|---|---|---|---|---|---|---|---|---|---|---|---|---|
| Morphine | | | | | Oxycodone | | | | | Diamorphine | | Alfentanil | | Fentanyl | Buprenorphine | Tramadol | Codeine Phosphate |
| Oral (mg) | | | Subcutaneous (mg) | | Oral (mg) | | | Subcutaneous (mg) | | Subcutaneous (mg) | | Subcutaneous (mg) | | Transdermal Patch **(mcg/hr). Stable pain only** | Transdermal Patch **(mcg/hr) Stable pain only** | Oral (mg) | Oral (mg) |
| 4 h dose (IR) | 12h dose (IR) | **24 h total dose** | 4 h dose | **24 h total dose** | 4 h dose (IR) | 12h dose (IR) | **24 h total dose** | 4 h dose | **24 h total dose** | 4 h dose | **24 h total dose** | 4 h dose | **24 h total dose** | **Change every 72 hours** | **Change at intervals indicated** | **24 h total dose** | **24 h total dose** |
| 1.25 | | **10** | | | | | | | | | | | | | 5 7 days | 100 | 120 |
| 2.5 | 10 | **20** | 1.25 | **10** | 1.25 | 5 | **10** | 1.25 | **5** | 1.25 | **5** | 0.125 | **0.5** | | 10 7 days | 200 | 240 |
| 5 | 15 | **30** | 2.5 | **15** | 2.5 | 10 | **20** | 1.25 | **10** | 1.25 | **10** | 0.125 | **1** | 6-12 | 15 7 days | 300 | |
| 7.5 | 20 | **40** | 5 | **20** | 5 | 10 | **20** | 2.5 | **10** | 2.5 | **10** | 0.25 | **1.5** | 12 | 20 7 days | 400 | |
| 10 | 30 | **60** | 5 | **30** | 5 | 15 | **30** | 2.5 | **15** | 2.5 | **15** | 0.25 | **2** | 12-25 | 35 72 h | | |
| 15 | 45 | **90** | 7.5 | **45** | 7.5 | 25 | **45** | 3.75 | **25** | 3.75 | **25** | 0.5 | **3** | 25-37 | 52.5 72 h | | |
| 20 | 60 | **120** | 10 | **60** | 10 | 30 | **60** | 5 | **30** | 5 | **30** | 0.75 | **4** | 37-50 | 52.5 72 h | | |
| 30 | 90 | **180** | 15 | **90** | 15 | 45 | **90** | 7.5 | **45** | 7.5 | **45** | 1 | **6** | 50-75 | | | |
